# Integrating Climate Change Adaptation into Disaster Risk Reduction in Urban Contexts: Perceptions and Practice

**DOI:** 10.1371/currents.dis.7bfa59d37f7f59abc238462d53fbb41f

**Published:** 2014-01-15

**Authors:** Claudia Rivera

**Affiliations:** Universidad Nacional Autónoma de Nicaragua (UNAN-Managua)Lund University

## Abstract

This paper analyses the perceptions of disaster risk reduction (DRR) practitioners concerning the on-going integration of climate change adaptation (CCA) into their practices in urban contexts in Nicaragua. Understanding their perceptions is important as this will provide information on how this integration can be improved. Exploring the perceptions of practitioners in Nicaragua is important as the country has a long history of disasters, and practitioners have been developing the current DRR planning framework for more than a decade. The analysis is based on semi-structured interviews designed to collect information about practitioners’ understanding of: (a) CCA, (b) the current level of integration of CCA into DRR and urban planning, (c) the opportunities and constraints of this integration, and (d) the potential to adapt cities to climate change. The results revealed that practitioners’ perception is that the integration of CCA into their practice is at an early stage, and that they need to improve their understanding of CCA in terms of a development issue. Three main constraints on improved integration were identified: (a) a recognized lack of understanding of CCA, (b) insufficient guidance on how to integrate it, and (c) the limited opportunities to integrate it into urban planning due to a lack of instruments and capacity in this field. Three opportunities were also identified: (a) practitioners’ awareness of the need to integrate CCA into their practices, (b) the robust structure of the DRR planning framework in the country, which provides a suitable channel for facilitating integration, and (c) the fact that CCA is receiving more attention and financial and technical support from the international community.

## Introduction

There is increasing recognition that disaster risk reduction (DRR) should include climate change adaptation (CCA) 1. CCA and DRR have been developed by different communities, but the aim of both is to reduce vulnerability and hazard exposure in order to increase resilience to the potential adverse impacts of climate extremes 2. Both DRR and CCA require collaborative and coordinated actions 3. The integration of the two fields provides opportunities to strengthen the common parts and improve the management of present and future hazards and risks 4. Moreover, it is commonly accepted that development and sustainable goals may be facilitated by integrating CCA into DRR 5. Just as importantly, the lack of integration of these fields will lead to redundant and conflicting responses 6.

The need to address DRR and CCA simultaneously in order to achieve coordinated actions has been stressed by both UNISDR 7 and IPCC-SREX report 2 . The latter states, for instance, that “countries more effectively manage risks if they include considerations of disaster risk in national development and sector plans and if they adopt climate change adaptation strategies, translating these plans and strategies into actions targeting vulnerable areas and groups” (p.10).

The need for integration of CCA is especially urgent in cities. The risk in urban areas is, for instance, aggravated by the fact that cities concentrate population, economic activities and built environments 8. The population of cities is constantly increasing, and if risk management is not taken into consideration in urbanization processes the risks will also increase 9. Thus, considering urban planning in the process of integration of CCA into DRR is a matter of urgency, not only due to the fact that vulnerabilities of cities need to be addressed, but also urban risk management is a potential entry to CCA and DRR 3.

Against this background, the purpose of this study was to describe the extent to which DRR practitioners are taking into consideration the creation of synergies and coordination between CCA and DRR. More specifically, this paper attempts to answer the question, “*How do disaster risk reduction practitioners perceive the on-going integration of climate change adaptation into their work on urban development?*” The integration of CCA into the policies and regulatory frameworks of DRR, the environment and urban planning in Nicaragua was analysed in a previous, quantitative, study 11. The purpose of the present, qualitative, study of the integration process is to complement this previous study.

The country studied is Nicaragua, which has a long history of dealing with disasters. Considerable amounts of knowledge and experience have been gained, and important advances have been made, in implementing DRR in Nicaragua, as well as in other Central American countries 12 ^,^ 13. Exploring how DRR practitioners deal with the integration of CCA provides valuable information on how CCA can be improved and on factors that may be limiting its implementation 14. Also, understanding the constraints of adaptation, and its integration process, contributes to the ability to assess, and consequently improve, decision-making processes 15.

The paper is organised as follows: Section 2 describes the background of the case study, Section 3 presents the conceptual framework, Section 4 presents the methods used and Section 5 presents the results and the analysis. Finally, the results are discussed in Section 6 and the conclusions are presented in Section 7.

## Background

After the devastation in Central America caused by the hurricane Mitch in 1998, national and international actors have been working on building capacities at all levels of government in order to reduce vulnerabilities in Nicaragua 16. The response of the Nicaraguan Government was to pass Law 337, which created the National System for Disaster Management and Prevention (SINAPRED in Spanish). Nowadays, this is the governmental body in charge of coordinating all DRR actions in the country. This system works in a top-down structure that coordinates all the institutions (government, non-government and private institutions) in the country that follow the decision-making process of the national committee formed by the authorities at the national level 17.

The subject of climate change was introduced in Nicaragua after the United Nations Framework Convention on Climate Change (UNFCCC) in 1992. The Kyoto protocol was ratified in 1999 by the Nicaraguan National Assembly. Since pledging to adhere to these international agreements, the national authorities have proposed climate policies and strategies 48. The National Strategy on climate change is managed by a top-down structure, where the decision-making process has three levels: the creation of legislation by the National Assembly, its implementation by the Ministries and their territorial delegations, and its management by the Ministry of Environment and Natural Resources (MARENA in Spanish) 17.

## Conceptual framework

There is an increasing amount of literature that identifies links, and the need to create synergies between CCA and DRR (e.g. 1 ^,^ 5 ^,^ 24). The IPCC-REX report 2  and the “Implementation of the Hyogo Framework for Action” 7 (UNISDR 2013) both strongly encourage the actors from both fields to coordinate their actions more closely. Each of these fields has different concepts and approaches that provide important inputs to the knowledge base on how to deal with climate-related events 2 (IPCC 2012). Descriptions of the most important concepts from both fields, used in this paper, are presented below.

Significant experience in dealing with disasters has been achieved in the field of DRR 25. DRR was established as a conceptual and operational approach to reduce the risk of disasters through systematic efforts to analyse and manage causal factors of disasters and risk, and includes the reduction of hazard exposure and the reduction of vulnerability of people and property 26. Substantial efforts have been made within this field to reduce the impact of both natural and manmade disasters on people and their livelihood 1.

Disasters are exacerbated by the effects of climate change, which will continue to affect the goals of achieving sustainable development 2 ^,^ 27. One of the latest definitions of climate change, published in the IPCC-SREX report 2, was “a change in the state of the climate that can be identified by changes in the mean and/or the variability of its properties and that persists for an extended period, typically decades or longer. Climate change may be due to natural internal processes or external forces, or to persistent anthropogenic changes in the composition of the atmosphere or in land use”. Human and natural systems can respond to climate change by adapting to its impacts 27. CCA is a process and related actions aimed at reducing the vulnerability of systems (e.g. urban systems) to the adverse impacts of anticipated climate change 20.

The fields of CCA and DRR have significant overlaps in managing risk to development 6 ^,^ 28. The main overlaps are: (a) sharing the same aim of reducing the effects of climate-related disasters and associated risks; (b) common stakeholders; (c) activities and measures for addressing climate-related disasters at household or community level 29.

Mainstreaming CCA into DRR is, thus, important in order to take actions to reduce the impact of extreme events 23. In this paper, mainstreaming is used to describe a specific way of integrating CCA into DRR. The term mainstreaming generally signifies the modification of a specific type of core work (such as urban planning) in order to take a new aspect or topic (such as CCA) into account, and to act indirectly upon it 29 ^,^ 30 ^,^ 31. It does not mean completely changing an organisation’s core functions or responsibilities, but instead viewing them from a different perspective and carrying out any necessary alternations. It is about looking into what already exists and building as much as possible on existing structures, mechanisms and procedures 29.

The integration of CCA and DRR must take into consideration aspects that will reduce vulnerabilities in cities because it is here a large proportion of those at risk from the effects of climate change are to be found 20. Thus, mainstreaming CCA in urban planning may allow coordinated and strategic actions to avoid the creation of unmanage­able levels of risk to a city’s built environment and population 9. In this paper, urban planning is defined as a discipline and a practical way of shaping and modifying urban settlements and space 18.

There is an increasing amount of literature containing recommendations and approaches for mainstreaming CCA into sustainable development and DRR (e.g. 32 ^,^ 21 ^,^ 33 ^,^ 34 ^,^ 35 ^,^ 36). The main­streaming process depends on the specific decision-making settings in different contexts 33. The approaches are not identical, but they share some aspects that may support the mainstreaming of CCA into DRR practice. Some studies are focused on overcoming barriers to the mainstreaming of CCA (e.g. 15 , 38), and propose processes related to mainstreaming actions during the planning phase (e.g. 32 ^,^ 33 ^,^ 36). Other studies are focused on mainstreaming CCA at the project level (e.g.^21^^,^ 33 ^,^ 34 ^,^ 35). Some of the recommendations suggested to have the potential to improve the process of mainstreaming in practice are given in Table 1 below.

**Table 1. Approaches for mainstreaming CCA:** 

Approaches	References
a) Understanding CCA in its political, institutional and government contexts. Practitioners at organisational level must also be aware of the activities of their organisations and relation with CCA.	CARE 2009, OECD 2009, Harris and Bahadur 2011, UNDP-UNEP 2011, Wamsler et al. 2013
b) Understanding the international and national regulatory and political frameworks related to CCA. This approach encourages practitioners to revise their plans, programmes and activities, and their connections with CCA, and to assess how their current and future programmes can be affected by climate change.	Mitchell, Tanner et al. 2006, CARE 2009, OECD 2009, Harris and Bahadur 2011, Wamsler et al. 2013
c) Evaluation and strengthening of institutional capacity to create tools to mainstream CCA and allocate resources.	Mitchell, Tanner et al. 2006, CARE 2009, UNDP-UNEP 2011, Wamsler et al. 2013
d) Engaging stakeholders and building partnerships with government and non-governmental actors at all levels in order to create or improve their degree of coordination.	Mitchell, Tanner et al. 2006, Harris and Bahadur 2011, UNDP-UNEP 2011, Saito 2013, Wamsler et al. 2013
e) Influencing the decision-making process and developing CCA measures.	UNDP-UNEP 2011, Wamsler et al. 2013
f) Improvement of monitoring systems for the mainstreaming process.	Moser and Ekstrom 2010, Harris and Bahadur 2011, UNDP-UNEP 2011, Saito 2013, Wamsler et al. 2013
g) Learning through experience obtained from the implementation of CCA measures at local level.	Mitchell, Tanner et al. 2006, Harris and Bahadur 2011, UNDP-UNEP 2011, Saito 2013, Wamsler et al. 2013

## Methods

The analysis in this study is based on qualitative semi-structured interviews. This method provides the opportunity to identify the meanings people attribute to their experience 39. The semi-structured interviews used in this study explore the reflections of the practitioners on the extent to which CCA is integrated into their practice of DRR in urban areas. As a way to operationalize the research question, the interviews were designed to collect individual-level information according to five specific aspects: (a) understanding CCA, (b) links between CCA and DRR, (c) links between CCA and urban planning, (d) potential measures to adapt cities to climate change, and (e) obstacles, gaps and opportunities for linking CCA with DRR and urban planning.

Purposive and snowball sampling were used for the selection of respondents. The former was used initially for the selection of respondents, to identify individuals that have knowledge of, or work in, the fields under study 40. The “Virtual Library of Disasters” 22 (CIES - UNAN 2005) was used for guidance. This is a website that has a list of the institutions involved in DRR in Nicaragua. The snowball sampling method was used during the development of the interviews, by asking for the names of other people who knew about the topic, and who worked in the same field 19. After the interviews, the practitioners that participated were classified into three groups according to the type of organisation in which they worked: government organisations, non-government organisations (NGOs) and universities. Although the public universities included in the third group belong to the government, their work and coordination is quite different from that of the government organisations covered in this study.

Nine interviews were conducted, including three operational officers, three academic staff and three programme managers (Table 2)^[1]^. Two of these interviewees also work in SINAPRED. Three of the respondents were from international NGOs, and four were from public universities.

**Table 2. Respondents of the semi-structured interviews:** 

Institution	Type of staff	Type of institution	Level
SINAPRED	Operational officer	Government	National
SINAPRED	Operational officer	Government	National
DIPECHO- European Commission	Programme manager	NGO	Regional
Habitat for Humanity	Programme manager	NGO	Local
Programme for Climate Change Technology Transfer Centers in Europe and Latin America	Academic	NGO	National
Multidisciplinary Regional Faculty of the Autonomous University of Nicaragua (FAREM-UNAN)	Academic	University	National
National University of Engineering (UNI)	Programme manager	University	National
Cleaner Production Center of Nicaragua (CPML – UNI)	Operational officer	University	National
Programme for Science and Technology for Development (PROCYTED – UNI)	Academic staff	University	Nationa

Transcriptions of the interviews were examined, and segments of data were classified into six categories^[2]^, using the content analysis method 41, using keywords to identify sections of texts that provided information about the relation of each field to the others, the nature of the connection between them and synergies. The following six categories were used:

- (A1) CCA: the understanding of CCA

- (A2) CCA-DRR: the links between CCA and DRR

- (A3) CCA-Urban planning: the links between CCA and urban planning

- (A4) CCA measures: potential adaptation measures for urban areas

- (A5) Obstacles/gaps: aspects that hinder the integration of CCA and DRR

- (A6) Opportunities: opportunities to improve the integration of CCA and DRR

Section 4 presents the results of this analysis using examples of the most relevant segment of data from the transcribed interviews.

Footnotes of the section:

****^[1]^**** *Only a limited number of participants were included, but they provided the perspective of the most important groups of practitioners in the country: governmental, non-governmental and academia. The study depended on their willingness to participate and their working agenda. One limitation of this study was confusion by some respondents when recommending other professionals that were not involved in the fields being studied. This can be interpreted as a finding in itself: i.e. that it is difficult for some practitioners to identify professionals working in CCA. Also, a theoretical saturation has occurred when no major new finding was gained and the latest respondents provided similar answers than the previous ones. However, the participants included in the study provided important clues on how integration is being dealt with.*

***^[2]^** These six categories were also used by Rivera and Wamsler (2014)*11* to explore the integration of CCA into the policy and regulatory frameworks of both DRR and urban planning in Nicaragua.*

## Results and analysis

**Understanding climate change adaptation**

During the interviews, the respondents were asked to explain their understanding of CCA. The key patterns identified were:

- CCA is key for sustainable development,
- CCA is a mainstreaming issue,
- CCA is under the control of environmental and technical institutes in the country, and
- there is a lack of understanding of how to mainstream CCA in practice.

All the respondents said that CCA is important for sustainable development, and they all considered that CCA is a mainstreaming issue in all working sectors of the country. However, they also expressed the opinion that CCA has been managed by technical institutions. They said that most CCA strategies and plans proposed by the government have been focused on environmental issues. One respondent expressed the following views:

“(…) there are institutions that think that climate change is exclusively for technical organisations. They do not see it with a multidisciplinary and institutional vision because climate change is not only about the atmosphere (…) it is about health, food security, productivity. I mean, there are many aspects about CCA that are not clear for many institutions, not yet (…)”^[i]^

The majority of them recognised that it is often not clear how measures for reducing the impact of climate-related events can be introduced into their practice, and in all the sectors of the country. They said that improving the understanding of the impacts of climate change in the DRR community and among urban planners, would be one important way to improve the integration of CCA.

**Climate change adaptation and disaster risk reduction**

The identified key patterns of the respondents’ understanding of the links between CCA and DRR were:

- CCA forms part of DRR actions,
- DRR and CCA are not different fields of work,
- the integration of CCA into DRR has started, and is currently focused on the creation and modification of policy frameworks and existent DRR methodologies, and
- universities are seen as potential driving forces to further integrate CCA into DRR, urban planning, and other areas.

All the respondents said that CCA is part of DRR, since climate-related events are included in DDR activities for managing risks. Furthermore, one respondent expressed the opinion that CCA and DRR converge in the same objective and that, in some way, they are working on the same actions but in different fields. The respondent said:

“(…) these two worlds have not yet found how to avoid doing the same things, but with different names, … in the end, we employ climate change adaptation, but it is included in disaster risk reduction. Full stop! (…)”^^[ii]^^

Two of them expressed the opinion that there is no difference between CCA and DRR. Thus, they said that climate-related risks should be dealt with as part of DRR, and it is not necessary to manage the same risks using two different frameworks. One of these respondents said that managing CCA and DRR in different structures would create confusion and the duplication of actions.

“(…) conceptually, there is no difference between traditional disaster risk reduction and climate change adaptation. Climate change is a risk just like any other. Perhaps the origin …, some risks might arise from natural hazards, and this is about exacerbated hazards caused by human beings (…)”^^[iii]^^

The respondents were asked to give examples of how they link the two fields in their work. The most concrete example was given by the respondents from the government organisation. They said that the integration of these fields has started by improving the current regulation system and creating projects that include issues from both fields. They explained that there are many aspects where CCA is integrated into the DRR framework at regional and national level. They specifically mentioned three ways in which this is achieved. The first is related to the approval of the “Policy on Comprehensive Disaster Risk Management in Central America”^[3]^ (PCGIR in Spanish) in 2010, which includes a programmatic area focused on CCA. The second is through the “National Policy of Disaster Risk Reduction” to be approved by the National Assembly. They explained that this policy includes a chapter on CCA. Finally, methodologies for DRR plans at local level are modified by adding CCA aspects.

The respondents from the universities stated that some departments are working on DRR educational projects, while others did not have any specific DRR programmes. One respondent from the National University of Engineering (UNI) said that there was a lack of departments at the university working on disaster risk reduction or climate change. However, some professors are very interested in the topics, and they are developing studies in this area, based on their own initiatives and involving some students.

All the respondents from the universities said that CCA could be easily integrated into their universities’ curricula, and that this would facilitate mainstreaming. For instance, the respondent from the National Autonomous University of Nicaragua (UNAN) described a project^[4]^ supported by the Swiss Development Cooperation and the NGO German Agro Action. The objective of this project is to train staff from universities, local government and NGOs in DRR. The project provides knowledge and methodological tools for integrating DRR into practice and improving actions in this field. CCA is one of the aspects included in the training material.

“*(…) we work on the training of technicians from institutions such as municipalities and other governmental organizations and NGOs. So, once they acquire the knowledge, they can apply it locally in preparation for disaster risk reduction (…)”^[iv]^*

*Footnotes of the section:*

^[3]^ The PCGIR is available at: http://www.sica.int/busqueda/Centro%20de%20Documentaci%C3%B3n.aspx?IDItem=44921&IdCat=32&IdEnt=22&Idm=1&IdmStyle=1

^[4]^ FAREM-UNAN/Estelí coordinates the project “Center for continuing education and training on risk management and disasters” http://www.farem.unan.edu.ni/riesgo/index.html.

**Climate change adaptation in urban planning**

All the respondents expressed the opinion that managing risks in cities is highly important. The key patterns of the respondents’ understanding of the integration of CCA in urban planning are:

- the integration of CCA into urban planning is important to address the increasing impact of climate change on cities,
- few practitioners include CCA in urban planning, and
- very limited advances have been made in urban CCA due the lack of instruments and institutional capacity.

The majority of respondents said that the areas of the country most affected by climate-related events are the largest cities, such as Managua and Estelí. They expressed the opinion that managing risks in cities is important in order to reduce the impact of climate change. A respondent from a government organisation said that land use planning and urban planning are aspects that must be considered in prevention planning. He also said that climate change could not be addressed if the urban contexts were not included in the analysis.

The respondent from the European Commission explained that after long experience of working in rural areas it was realized that DRR is also important in urban centres. For this reason, 75% of their current projects in Central American are focused on urban contexts. This respondent also explained that they are working on promoting DRR at local level through the campaign “Making Cities Resilient”^[5]^ of the United Nations Office for Disaster Risk Reduction. Within the frame­work of this campaign, they are working on training and increasing awareness of disaster risk reduction among the cities’ mayors.

Most of the respondents think that only a few practitioners and instruments are focused on managing CCA in urban areas. Two respondents said that local governments have the opportunity to improve DRR actions at local level and to integrate CCA measures in urban areas. However, they said that the few projects that could integrate CCA into urban planning were not noticeable. One respondent said:

“(…) there have only been a few people working in this area (CCA). I mean, even less than the ones involved in disaster risk reduction, and even less in urban scenarios, but the few people involved have not had the capacity to influence the rest to create a culture in their institutions…at least at the level I participated in or knew (…)”^^[v]^^

Also, the majority of respondents said that the integration of CCA in urban contexts is not having a real impact because of the lack of instruments and capacities of local and national governments. There was a general understanding that improvement of the instruments and regulations concerning urban planning could contribute to the reduction of disasters, including those caused by climate change.

*Footnotes of the section:*

^[5]^ http://www.unisdr.org/campaign/resilientcities/

**Climate change adaptation measures in urban areas**

To investigate the understanding of current and potential linkages between CCA and urban planning, the respondents were asked to give examples of potential measures that could be used to adapt cities to climate change. They suggested physical and non-physical measures that have potential for adapting urban areas to expected events resulting from climate change (Table 3). They were also asked to describe measures that they had already applied in their programmes. They proposed adaptation measures related to the expected impacts of climate change, such as extreme temperatures, urban droughts, sea and lake level rise, and floods. One respondent had published a book, “Notes on Climate Change in Nicaragua”^42^ and referred to the CCA measures included there (Table 3).

**Table 3. Measures for adaptation to climate change in urban areas identified by the respondents:** 

**Physical measures**	
Current status	Measure
Implemented	Relocation of vulnerable settlements. Housing improvement programmes do not include complete risk assessment studies, but they take into consideration factors that may cause the exposure of existing and new settlements to risks (e.g. proximity to water bodies). Policies for housing improvement programmes include the protection of vegetation. The replacement of trees and gardens, where necessary, is included in the technical assessment. The housing programmes include the improvement of water management systems, including waste water management.
Proposed by respondents	Promotion of mud roofing tiles in order to reduce the temperature inside houses. Preservation and promotion of the tradition of having trees in backyards and gardens to contribute to water infiltration. Promotion of green areas in cities in order to reduce run-off water. Use of native plants that need less water and maintenance in green areas of cities.* Improvement of indoor comfort by using windows for cross ventilation.* Building orientation according to the path of the sun in order to decrease the temperature.* Ensuring that the distance between buildings is greater than 0.6 m.* The use of light colours on building facades to reflect heat and sunlight.*
**Non-physical measures**	
Current status	Measure
Implemented	Monitoring of highly vulnerable locations to prevent relocated populations from moving back. Improvement of the early warning system designed for climate events.
Proposed by respondents	Creation of campaigns for cleaning and avoiding garbage in drainage systems in order to avoid clogging. Rainwater collection for irrigation of green urban areas.*
* Milan Pérez (2009)^42^	

**Opportunities and constraints on integrating climate change adaptation**

During the interviews, the respondents expressed their opinions about the importance, gaps, obstacles and opportunities for the integration of CCA into the fields of DRR and urban planning.

**Opportunities**

The patterns of the respondent’s understanding of the existing opportunities for further integration identified were:

- recognition of the importance of integrating CCA for sustainable development,
- the existing national DRR structure may contribute to improving the integration of CCA,
- the decentralized work of local governments would allow better integration, in accordance with local conditions,
- universities are suitable organizations to improve knowledge and create capacities, and
- CCA is an attractive concept to obtain financial and technical support.

All the respondents said that CCA must be included in any developmental action. It is an aspect of mainstreaming that should be considered in all sectors of the country. One respondent said that the need to consider CCA and DRR always comes across in any study that they conduct, although the aim of their research was not related to this topic.

The government staff considered that the existing structure of SINAPRED provides advantages for integrating CCA. They suggested that the current instruments and tools could be extended, and that these new aspects could be added. They also said that the communication channels they have with all the institutions in the country may be useful to reach the actors concerned.

One respondent mentioned that the autonomy of local governments, in creating plans and managing their own budgets, and their responsibility to establish urban development plans is suitable for integrating CCA into urban planning, according to local conditions.

Most of the academic respondents said that promoting and supporting better training of university staff in climate change and climate change adaptation would allow the transfer of knowledge to future practitioners.

The respondents pointed out that there are many opportunities for obtaining funds for projects focused on climate change from international sources. They expressed the opinion that CCA is attracting the interest of important donors that are willing to help by providing financial and technical support. They mentioned that this is an important incentive for the integration of CCA. One respondent also added that CCA is attractive not only in the public sector, but also in the private sector. If there is awareness among practitioners of the benefits of adapting to and mitigating the effects of climate change, its integration into DRR and urban planning would be improved.

**Constraints**

The patterns of the respondent’s understanding of the existing constraints on further integration identified were:

- poor understanding of CCA,
- CCA being mainly managed by environmental institutions, and
- the lack of connections between universities and other institutions.

The main gaps identified by the respondents concern knowledge and the understanding of CCA. They said that there is confusion regarding the concept of CCA. Two respondents claimed that many practitioners do not understand climate change and its implications. They said that CCA is fragmented between many actors and institutions, and they have not yet reached a consensus regarding concepts and actions.

The respondents said that today, CCA is mainly managed by actors concerned with environmental protection and food security. They argued that CCA must also be promoted in cities by its integration into urban planning, supported not only by technical institutions, but also by all Ministries. However, the focus on environmental issues seems to limit the integration of CCA into other areas of development.

The interviewees expressed the opinion that the new generation of practitioners is not learning modern methodologies and concepts derived from experience due to a lack of cooperation between the institutions working on DRR and universities.

## Discussion

This study provides important insights into how the integration of CCA into DRR is hindered, and how it could be improved. The results indicate that most of the identified constraints on the integration of CCA into DRR are related^17^ to the level of knowledge of practitioners concerning CCA. The information obtained from the respondents in the present study concerning their understanding of CCA and its influence on integration can be categorized into three barriers at the stage of understanding, based on the classification of Monser and Ekstrom (2010)^15^ .

The first barrier at the stage of understanding is related to: “some actors are too distant to the signal to take note” 15 (p.3). The respondents expressed the opinion that CCA has been an issue that has mainly involved environmental and technical institutes. This perception coincides with the process of integrating CCA into policies and regulatory frameworks in the country, in which CCA was managed exclusively by these kinds of institutions 17. This action contributes to creating the conception that CCA must be managed by scientists, rather than being a problem related to development 25. It also reveals inadequate “deficit models” 43, where technical and scientific knowledge on CCA is communicated in a top-down structure, using complex language that hardly addresses the common understanding of science. Practitioners obtain most of their information from the authorities through official channels. For this reason, non-scientific practitioners are not encouraged to include CCA in their practices.

A second barrier identified at the stage of understanding is the uncertainty and variability of climate change: “that the signal does not clearly emerge from the background noise” 15 (p.3). Most of the respondents showed uncertainty when discussing the integration of CCA into their practices. However, they are aware of their limited understanding about CCA, and how to deal with expected climate-related events using non-technical and non-scientific approaches. As Gifford (2011)^44^ states, the lack of knowledge allows the creation of a gap between attitude and behaviour. Most of the respondents showed a positive attitude towards CCA, and they recognized how its management in urban contexts would reduce damage and loss arising from expected climate-events. However, it was difficult for them to identify direct links between CCA and their practices.

The unclear understanding of CCA has also discouraged practitioners from mainstreaming CCA into DRR. For instance, two respondents stated that instead of considering integration between the two fields, they tended to ignore CCA. One of the reasons for this is that DRR has been strongly promoted and supported by national and international cooperation. As a result, practitioners had a comprehensive understanding of DRR. Thus, the actions taken in the framework of DRR are clearer than the uncertain issues of social responses to climate change 45.

Another aspect that increases the uncertainty concerning climate change is the conception that it is a long-term risk. Practitioners expressed their worry about natural events that they perceived as being more hazardous in the short term, such as seismic activity. As pointed out by Weber (2006)^46^ , actors often express their concern and focus their attention on situations that they feel are likely to materialize. This was the case for most of the practitioners interviewed in the present study.

The third and final barrier identified at the stage of understanding is the lack of guidance, which can determine the capacity and willingness to make decisions regarding CCA 15. A positive aspect related to this barrier, is that the majority of respondents showed an interest in learning about and adopting CCA measures in their work. However, they also identified a lack of proper guidance in dealing with climate change and how mainstreaming CCA into their DRR practices.

Regarding the integration of CCA into urban planning, most of the respondents stated that CCA was not integrated into urban contexts. They mentioned three barriers that hinder the integration of CCA into urban planning. The first is the lack of comprehensive, up-to-date policies and instruments for urban planning. The most relevant documents in the regulatory framework for urban planning in Nicaragua are from 1982, and have not been updated. The second is the complexity of the current urban system, which limits opportunities to create urban processes and adaptive responses that contribute to sustainability in cities. The growth of cities in Nicaragua, mainly Managua, has been the result of urban sprawl for many decades 47. This situation also contributes to the third barrier, namely inadequate urban planning practice in Nicaragua, mostly due to the reasons discussed above.

From the above, it follows that the lack of up-to-date regulatory planning policies and regulations that has been identified needs to be addressed simultaneously alongside the integration of CCA, with special consideration given to the particularities of urban areas. Urban risk and disasters “are unique in the sense that they occur in an environment that has adapted to absorb large population and services leading to specific characteristics related to: (a) scale, (b) densities, (c) inhabitants’ livelihood strategies, (d) economic systems and resource availability, (e) governance systems, (f) public expectations, (g) settlement structures and form, (h) likelihood for compound and complex disasters, and (i) potential for secondary impacts on surrounding rural areas and regions”^29^ (p.4). These aspects thus need to be taken into consideration in the integration of CCA into urban planning.

Another finding is that the practitioners who were interviewed revealed that the integration of CCA in practice has reached different levels in DRR and urban planning. The results of this study showed that practice is closely linked to the development of policies and regulatory frameworks. The integration of CCA into policies and regulatory frameworks for the environment, DRR and urban planning in Nicaragua is a gradual process, subject to on-going modifications, which started from a very restricted focus on the protection of natural resources and food security, and developed into the current interest in creating comprehensive approaches to adaptation and risk reduction planning 11. This study shows that the integration of CCA into urban planning practice is also very limited.

Most developing countries, including Nicaragua, are facing several barriers for the integration of CCA into practice 2. Effective integration of CCA requires inter-sectoral and participative work that includes stakeholders and practitioners at national and local level as well as related monitoring and learning mechanisms. The policies and regulatory frameworks in Nicaragua are relatively recent 11, and continuous updates and modifications offer new opportunities for the integration of CCA.

Although the integration of CCA seems to be at an early stage, especially in urban planning, the practitioners identified opportunities that have the potential to promote it. They believe that CCA is attractive for obtaining technical and financial support from international aid agencies. Furthermore, practitioners from government organisations expressed the opinion that the channels of communication in the existing structure for DRR provide an easy way of integrating knowledge and actions concerning CCA.

## Conclusions

*The integration of CCA into current practices in urban contexts in Nicaragua is at an early stage.* DRR practitioners are aware of the need to improve their knowledge, and of the importance of adapting cities to climate change. Although they tend to leave the management of CCA to environmental and technical institutions, most of them expressed their interest in achieving a better understanding of CCA and of becoming actively engaged in the mainstreaming of CCA into their work.

*The main barrier is the perceived lack of understanding of CCA.* The outcome of this study suggested that this barrier could be overcome by creating professional education programmes and designing better communication strategies between the scientific community working on CCA and other non-scientific actors. Involving practitioners and policy makers in the creation of climate change scenarios and raising awareness about the impact of climate change in the national context would encourage them to find ways to mainstream CCA into their work.

*In addition, the study revealed that the progress of integration is closely linked to the improvement and creation of policies and regulatory frameworks. *The practitioners showed how receptive they are to international agreements and instruments. The practitioners in government organisations expressed their interest in increasing CCA integration into their practice because of the importance of fulfilling international and regional commitments. The stakeholders at national level were called upon to promote and contribute to CCA integration at policy level, and to enhance engagement among practitioners at all levels.

*The practitioners also expressed their confidence in the existing DRR system.* They said that the DRR framework in Nicaragua has a robust structure, and that they have achieved a comprehensive understanding of DDR. The creation of coordinated actions between CCA and DRR would avoid the duplication of effort, and ensure better use of human and financial resources. In order to do so, it is important to reach a consensus among practitioners for the creation of holistic approaches to the coordination of actions between CCA and DRR that need to be included in the policies and instruments of both fields.

*The mainstreaming process does not mean the creation of a new separate structure for CCA. *Instead, SINAPRED and the organisation that forms the DRR system need to explore the potential for improving the mainstreaming process by: a) reviewing and adapting existing and planned programmes in order to take CCA into consideration; b) by evaluating their institutional policies and capacity, to reduce internal vulnerabilities to climate change; c) strengthening of networks of complementary partners that provide different perspectives and approaches to address climate change; and d) creating mainstreaming monitoring and evaluation mechanisms that offer the opportunity to learn from experience.

*Today, CCA is poorly integrated into urban planning in Nicaragua.* The interviewees identified shortcomings and gaps in urban planning policies and instruments as one of the main problems. Urban CCA requires tools that can guide the day-to-day work of practitioners in cities at risk. It is important to consider the complex systems of cities and their influence on disaster occurrence; specifically, how cities can modify or exacerbate the characteristics of hazards, local vulnerabilities and the mechanisms for response and recovery (cf. 31 ). The revision and improvement of urban planning tools to include DRR and CCA as an integral part are crucial to create mutual integration of the three fields to, ultimately, protect existing urban societies and design resilient cities in the future.

## Competing Interests

The author has declared that no competing interests exist.

## Appendix 1

### 1. Original quotations

^ ^^[i]^ (…) hay instituciones que piensa que el cambio climático es sólo de las entidades científica técnica, y no lo ven como que deben tener una visión interinstitucional y multidisciplinaria porque el cambio climático nos solamente es la atmósfera (…) es la salud, la seguridad alimentaria, es la producción. O sea hay un montón de elementos -sobre la adaptación al cambio climático- que muchas instituciones no los tienen complemente claro aún (…)

^[ii]^ (…) ahora, esos dos mundos que todavía no hemos encontrado como no hacer lo mismo con nombres diferentes….al final, sí hacemos adaptación al cambio climático, pero está integrado en la operación (de gestión del riesgo) ¡Y punto! (…)

^ ^^[iii]^ (…) para mí, conceptualmente no hay ninguna diferencia entre la gestión de riesgo tradicional y la adaptación al cambio climático. – Cambio climático - es un riesgo como cualquier otro. Quizás la etiología aquí, hay riesgos que pueden ser de amenazas naturales y cambio climático es una amenaza exacerbada por el ser humano (…)

^ ^^[iv]^ (…) trabajamos en la capacitación de técnicos de las instituciones como la alcaldía y otras instituciones gubernamentales y no gubernamentales. Para que una vez que ellos adquieran el conocimiento, puedan aplicarlos en la preparación local para la gestión de riesgos (…)

^[v]^ (...) ha habido muy poca gente en el tema (adaptación al cambio climático). Que se haya metido en el tema de gestión de riesgos y los escenarios urbanos, menos todavía pero los pocos que se han metido no han tenido la capacidad de incidir sobre los demás de crear una cultura sobre las instituciones... por lo menos en los niveles donde he tenido alguna participación o he sabido (…)
